# MAP4 as a New Candidate in Cardiovascular Disease

**DOI:** 10.3389/fphys.2020.01044

**Published:** 2020-08-26

**Authors:** Lingfei Li, Qiong Zhang, Xia Lei, Yuesheng Huang, Jiongyu Hu

**Affiliations:** ^1^Department of Dermatology, Daping Hospital, Third Military Medical University (Army Medical University), Chongqing, China; ^2^Institute of Burn Research, Southwest Hospital, Third Military Medical University (Army Medical University), Chongqing, China; ^3^State Key Laboratory of Trauma, Burns and Combined Injury, Third Military Medical University (Army Medical University), Chongqing, China; ^4^Department of Endocrinology, Southwest Hospital, Third Military Medical University (Army Medical University), Chongqing, China

**Keywords:** microtubule-associated protein 4, microtubule-associated proteins, microtubule, mitochondria, cardiovascular disease

## Abstract

Microtubule and mitochondrial dysfunction have been implicated in the pathogenesis of cardiovascular diseases (CVDs), including cardiac hypertrophy, fibrosis, heart failure, and hypoxic/ischemic related heart dysfunction. Microtubule dynamics instability leads to disrupted cell homeostasis and cell shape, decreased cell survival, and aberrant cell division and cell cycle, while mitochondrial dysfunction contributes to abnormal metabolism and calcium flux, increased cell death, oxidative stress, and inflammation, both of which causing cell and tissue dysfunction followed by CVDs. A cytosolic skeleton protein, microtubule-associated protein 4 (MAP4), belonging to the family of microtubule-associated proteins (MAPs), is widely expressed in non-neural cells and possesses an important role in microtubule dynamics. Increased MAP4 phosphorylation results in microtubule instability. In addition, MAP4 also expresses in mitochondria and reveals a crucial role in maintaining mitochondrial homeostasis. Phosphorylated MAP4 promotes mitochondrial apoptosis, followed by cardiac injury. The aim of the present review is to highlight the novel role of MAP4 as a potential candidate in multiple cardiovascular pathologies.

## Introduction

Cardiovascular disease (CVD) is the leading cause of mortality worldwide, accounting for approximately 40% of all deaths (Schwalm et al., 2016). Hypoxia/ischemia, hypertension, inflammation, diabetes, cardiomyopathies, cardiometabolic syndrome, and obesity are the common risk factors for CVD, and these multiple comorbidities elevate the rates of mortality and morbidity in CVD (Koene et al., 2016; Ceylan et al., 2018; Ren and Zhang, 2018; Zhang et al., 2018; Zhou et al., 2018a; Caporizzo et al., 2019). Despite the advent of a number of drugs and techniques, only limited efficacy has been reached in CVD treatment. Considering these facts, novel interventions, with a broad scope to cover these multiple comorbidities, are necessary.

Cardiovascular dysfunction leads to the development and progress of CVD. Cardiomyocytes and endothelial cells are mainly involved in the maintenance of normal cardiovascular function. Accumulating evidence suggests that damaged microtubules (MT) and mitochondria are the key players in the pathogenesis of cardiomyocytes and endothelial dysfunction. As an important cytoskeletal component, MTs are involved in cell death, maintenance of cellular homeostasis, and organelle transport, and the instability of MT dynamics induces cardiac hypertrophy/heart failure (Tagawa et al., 1996; Sato et al., 1997), myocardial ischemia–reperfusion injury (Sato et al., 1993), and catecholamine-induced myocardial injury. Since mitochondria are involved in cell metabolism, oxidative reactions, and energy supply, mitochondrial disruption reportedly leads to apoptosis, metabolic disorder, and excessive oxidative stress, which contribute to the dysfunction of cardiomyocytes and endothelial cells, and is followed by abnormal cardiovascular events (Bravo-San Pedro et al., 2017; Suomalainen and Battersby, 2018).

In the current review, we discuss a novel candidate intervention target in CVD and elucidate its evolutionary genetic history, basic characteristics, and function, and its correlation with MTs and mitochondria.

## Microtubule-Associated Protein 4 (MAP4)

### Gene Evolutionary History

In mammals, microtubule-associated proteins (MAPs) are mainly consisted of MAP2, tau (also known as MAPT), and MAP4, all of which are crucial cytosolic skeletal proteins. Both MAP2 and tau are mainly distributed in neurons and have been reviewed elsewhere (Dehmelt and Halpain, 2005). MAP4 is a unique member of MAPs that is widely expressed in various tissues and cells, and recently, an increasing number of studies have suggested that MAP4 might play an important role in cardiovascular disorders. MAP4 is encoded by a single gene located in 3p21 and consists of 23 exons. Moreover, MAP4 gene has many isoforms, as well as three to five MT binding repeats (Dehmelt and Halpain, 2005). Following isolation of the cDNA sequence encoding human and mouse MAP4, three distinct regions consisting of related sequences with different motifs were identified in its open reading frames. Analysis of its amino acid sequence suggests that human, mouse, and bovine MAP4 are close homologs and their sequences reach 0–75% similarity, and have highly conserved domains, including the amino terminus, MT binding domain, and carboxyl terminus, thus inferring that they are members of the same class (West et al., 1991).

### Characteristic Structural Features

The MAP4 protein is a ubiquitous, heat-stable protein composed of an asymmetrical structure similar to other MAPs and consists of an amino terminal projection domain and a carboxyl terminal MT binding domain (Mangan and Olmsted, 1996). Accumulating studies have shown that the MT binding domain contains a conserved phosphorylated KXGS motif (Lewis et al., 1988; Drewes et al., 1995; Illenberger et al., 1996; Ozer and Halpain, 2000). It has been reported that the phosphorylation sites, such as Ser696, Ser768, and Ser787 (corresponding to Ser667, Ser737, and Ser760 in mouse) in the proline-rich region of the human MT binding domains, are involved in the dissociation of MAP4 from MTs and regulate MT dynamics instability (Srsen et al., 1999; Kitazawa et al., 2000; Hu et al., 2010). MT dynamic is a process that depends on α and β subunits of tubulin, their post-translational modifications, and interaction of MT with MAPs, and is regulated by the balance between MT polymerization and disassembly, the disruption of which could lead to a series of diseases. Although a number of studies have concentrated on this region, the detailed mechanism of the regulation of MT dynamics remains unclear (Chapin and Bulinski, 1991; Tokuraku et al., 1999). In contrast, the amino terminal projection domain, which protrudes from the MT wall and does not bind to MTs, varies in length among different MAPs. The projection domain of MAP4 consists of three regions, including the amino terminal acidic region, the KDM-repeat sequence, and the b-regions. The region containing KDM-repeat sequence is a representative structure of the projection domain, and is a highly conserved unit composed of an imperfect 14 amino acid repeat (KD(M/V)X(L/P)(P/L)XETEVALA) starting with KD(M/V) (Aizawa et al., 1990; Chapin and Bulinski, 1991; Permana et al., 2005). Recent studies have indicated that the projection domain of MAP4 allows the separation of individual MTs by inhibiting the bundle-forming ability of the MT binding domain. Further, this suppressive activity of the projection domain is correlated with its length rather than its amino acid sequence. However, the detail process of the regulation of MT dynamics or their organization by the projection domain remains elusive (Faruki and Karsenti, 1994; Tokuraku et al., 1999; Iida et al., 2002; Permana et al., 2005).

### Distribution

Microtubule-associated protein 4 is reported to be widely expressed in non-neuronal cells and tissues, such as the adrenal gland, liver, lung, heart, vascular tissues, and skin (Parysek et al., 1985; Kotani et al., 1988; Matsushima et al., 2005; Hu et al., 2010; Li et al., 2015; Xia and He, 2018; Zhang et al., 2019a).

### Function

#### MAP4 and Cytoskeleton

Microtubules dynamics is affected by the balance between tubulin polymerization and depolymerization. As a cytoskeleton regulator, MAP4 regulates MT dynamics through its posttranscriptional phosphorylation modification (Chapin and Bulinski, 1991; Nguyen et al., 1997). Under normal conditions, MAP4 combines with MTs, resulting in the stability of MTs. Once phosphorylated, MAP4 dissociates from MTs, leading to MT disassembly and dynamic instability (Ebneth et al., 1999; Hu et al., 2014). Previous studies indicated that the MT binding domain of MAP4 contains a repeat region with tandemly organized repeat sequences, and different types of MAP4 contain a different number and arrangement of these repeat sequences. However, different isoform fragments of MAP4 exhibit similar degrees of MT polymerization activity and MT binding affinity, suggesting that variations in the repeat region are not essential for the regulation of MT dynamics, which is believed to be the main function of MAP4. In contrast, the MT bundle-forming activity is reported to differ among MAP4 isoform fragments, with the bundle formation being augmented by increasing the number of repeat sequences in the fragments, indicating that the role of MAP4 isoforms is to regulate the surface charge of MTs (Tokuraku et al., 2003). In addition, Tokuraku et al. (2003) demonstrated that MAP4 isoforms may also affect kinesin motor activity by regulating MT surface properties. They analyzed MAP4 isoforms in an *in vitro* gliding assay and found that the five-repeat isoforms suppressed the movement of MTs in a concentration-dependent manner. Furthermore, they revealed that MAP4 isoforms did not compete with kinesin for binding to MTs in a sedimentation assay, suggesting that kinesin could bind to MAP4-bound MTs, although it could not move on them (Tokuraku et al., 2007).

Semenova et al. (2014) used *Xenopus melanophores* to test the regulation of MT transport by MAP4. They found that pigment granules moved along the MTs to the cell center or periphery by means of dynein and kinesin-2, respectively. They showed that aggregation signals induced phosphorylation of threonine residues in the MT binding domain; meanwhile, the binding of the protein to MTs was shown to be reduced. Whereas overexpression of *Xenopus* MAP4 suppressed pigment aggregation by shortening dynein-dependent MT runs of melanosomes, removal of *Xenopus* MAP4 from MTs was reported to decrease the length of kinesin-2-dependent runs, inhibiting pigment dispersion. Thus, their data indicated that the MAP4-MT binding promoted the kinesin-2-based movement in parallel with the suppression of dynein-dependent movement of melanosomes. In addition, phosphorylation led to the dissociation of *Xenopus* MAP4 from MTs, thereby raising dynein-dependent and reducing kinesin-2-dependent motility of melanosomes, which stimulates their accumulation in the cell center. Conversely, dephosphorylation of *Xenopus* MAP4 during dispersion triggered the opposite effect (Semenova et al., 2014).

On a separate note, Matsushima et al. (2012) tested the interaction of MAP4 with actin filaments. Their data demonstrated that MAP4 and its MT binding domain fragments bound to actin filaments under normal conditions. The apparent dissociation constant and binding stoichiometry of the fragments to actin have been demonstrated to be approximately 0.1 μM and 1:3 (MAP4/actin), respectively, with the actin binding site on MAP4 reported to be located at the carboxyl terminal part of the proline-rich region, consistent with the MT binding site. Besides, they showed that MAP4-bound actin filaments tended to be straighter and longer, with the number of actin bundles increasing when the MAP4 fragment was added, suggesting that MAP4 binding altered the properties of the actin filaments. A multiple sequence alignment of the proline-rich regions of MAP4 and tau also revealed the presence of two putative actin-binding consensus sequences (Matsushima et al., 2012).

#### MAP4 and Cell Cycle

Previous studies demonstrated that mutations in human MAP4 (Ser696 and Ser787) affect MT dynamics and subsequently mediate the progression of cell cycle, with p34cdc2/cyclin B being deemed an important kinase in this biological process. In addition, the phosphorylation of the human MAP4 at Ser787 is noted during mitosis, whereas the phosphorylation of MAP4 at Ser696 is shown to occur throughout the cell cycle in proliferating cells. Overexpression of dephosphorylated MAP4 did not affect cell doubling time; in contrast, expression of phosphorylated MAP4 may affect progression into or through cell division, indicating that MAP4 phosphorylation plays a crucial role in regulation of cell cycle (Tombes et al., 1991; Ookata et al., 1995, 1997; Nguyen et al., 1997; Chang et al., 2001).

#### MAP4 and Mitosis

The MAP4 protein is involved in the regulation of MT dynamics during the M-phase (Ookata et al., 1995; Dehmelt and Halpain, 2005). However, both the MT network and MT dynamics show little change when MAP4 is removed from MTs using a blocking antibody. In addition, absence of MAP4 binding to MTs results in maintenance of the normal structure of spindles in cells progressing to mitosis, and posttranslational modifications of tubulin subunits also remain unchanged, suggesting that MAP4 may just be a component of a functionally redundant system (Wang et al., 1996). Zahnleiter et al. (2015) found that MAP4 mutations lead to the development of a clinical spectrum of centrosomal defects and demonstrated that centrosomal proteins play a regulatory role in the centrosomes and ciliary and Golgi bodies related to severe short stature. Both MAP4 and MT rescue factor and cytoplasmic linker associated protein 1 (CLASP1) are necessary for maintaining spindle location and an accurate cell division axis. In human cells, CLASP1 is required for capturing astral MTs, whereas MAP4 prevents engagement of excess dynein motors (Samora et al., 2011). Consistent with this, MAP4 was observed to interact with dynein-dynactin, thereby suppressing dynein-induced MT sliding. In contrast, absence of MAP4 leads to spindle disorientation in the vertical plane, revealing that force generators are under spatial control. Collectively, these data showed an essential role of MAP4 during mitosis, as spindle positioning is considered to be crucial during embryogenesis and stem-cell homeostasis (Samora et al., 2011). Shiina and Tsukita (1999) reported that mutations of serine and threonine to alanine at p34cdc2 kinase-specific phosphorylation sites interfered with the mitosis-associated reduction in MT affinity of *Xenopus* MAP4, and their overexpression affected the chromosomal movement during anaphase A in mitotic cells.

### Regulating Mechanisms

As already known, MT dynamics are affected by a variety of factors, including drugs (e.g., taxol and colchicine), temperature, GTP, free tubulin, and MAPs. Moreover, the effect of drugs and GTP on MT dynamics was also shown to be mediated by MAP4 (Holmfeldt et al., 2002, 2003; Delphin et al., 2012; Gombos et al., 2013; Ojeda-Lopez et al., 2014; Watanabe and Goshima, 2014).

Xiao et al. (2012) found that the MT binding domain of MAP4 binds to the outer and luminal surfaces of MTs, and taxol, an MT stabilizing drug, suppresses these interactions. Although taxol negatively regulated the interaction of MAP4 with MTs, its binding to the MT binding domain-MAP4-MT complex further decreased the overall deuterium incorporation, suggesting that a more stable complex was formed in the presence of the drug (Xiao et al., 2012). In addition, DNA-damaging agents could affect sensitivity to anti-MT drugs through the regulation of the expression of MAP4 (Zhang et al., 1998, 1999). Chapin and Bulinski (1994) revealed that MAP4 could bind to different types of MTs, including detyrosinated and tyrosinated tubulin, with the differential binding to these forms of tubulin not directly leading to a mechanism of segregation of MAP4 on MTs. Additionally, they also suggested that in TC-7 cells, the absence of MAP4 may affect rapid growth of MTs to which MAP4 was not yet bound, or the presence of other MAPs may compete with MAP4 for MT binding sites. These observations indicate that different binding states of MAP4 on MTs directly regulate MT dynamics within single cells, as well as other MT functions such as those involving MT motor activity (Chapin and Bulinski, 1994).

Septins are a conserved family of GTPases forming heterooligomers and interacting with the actin-based cytoskeleton and MTs. Ghossoub et al. (2013) explored the effect of septins in the primary cilium of retinal pigmented epithelial cells and showed that septin2, septin7, and septin9 formed a 1:1:1 complex, colocalizing along the length of the axoneme. Similarly, knockdown of cilium-localized septins were reported to suppress ciliogenesis in kidney epithelial cells. As a binding partner of septin2, MAP4 regulated the accessibility of septins to MTs and was also localized to the axoneme, where it appeared to negatively control ciliary length. This study provided new insights into the functions and regulations of septins and MAP4 in the organization of the primary cilium and MT-based activities in cells (Ghossoub et al., 2013). Kremer et al. (2005) found that reduced septin in Hela cells leads to a robust elevation in MT stability, and they also identified MAP4 as the septin binding partner using mass spectroscopic analysis. Septin2, spetin6, and septin7 were shown to directly bind to the proline-rich region of MAP4 to form a heterotrimer and this binding suppressed the ability of the MAP4 fragment to bind and bundle MTs. In addition, the absence of septin increased the number of abnormal cell, whereas this effect was suppressed when MAP4 was knocked down. Thus, these data reveal a novel role of septins in mediating MT dynamics through interaction with MAP4 (Kremer et al., 2005).

Hoshi et al. (1992) reported that MAP4 might be a good substrate for MAPK, with its phosphorylation leading to inactivation of tubulin polymerization. In addition, Mori et al. (1991) demonstrated that protein kinase C regulated the activity of MAP4 and subsequently led to changes in the MT dynamics.

## Microtubules in CVD

Microtubules, the major component of the cytoskeleton, are composed of α/β tubulin dimers and exhibit a crucial role in regulating many processes, including cell survival and death, cell migration and proliferation, cell shape maintenance, and organelle transport (Walczak, 2000; Birukova et al., 2004; Hu et al., 2010; Zhang et al., 2013, 2019a). In particular, MTs target focal adhesions to regulate extracellular matrix adhesion, and the attachment of MTs to special cortical regions of cells is required for cell polarization (Small et al., 2002). The functions of MTs are affected by MT dynamics as active polymerization and depolymerization indicate MT growth and shortening (Mitchison and Kirschner, 1984). Thus, the growth, shortening, catastrophe, and rescue of MTs are reflected by MT instability, induced cellular homeostasis, division, and movement. Based on the essential functions of MTs, many researchers have investigated the role of MT dysfunction in CVD, including cardiac hypertrophy/heart failure (Tagawa et al., 1996; Sato et al., 1997), myocardial ischemia–reperfusion injury (Sato et al., 1993), and catecholamine-induced myocardial injury (Hori et al., 1994). In addition, desmin intermediate filaments have been identified as a key component of an anchoring complex that links MTs to the sarcomere and imparts structural organization to the MT network. Mutations in the human desmin gene lead to an autosomal dominant, autosomal recessive, and sporadic forms of protein aggregation myopathies and cardiomyopathies (Clemen et al., 2015; Robison et al., 2016). Previous studies have also reported that a complex receptor dependent or independent kinase cascade regulates the transcription of MT deformation-sensitive genes, such as *PAI-1* and *CTGF*, which may provide novel targets to impede the development of CVD (Samarakoon and Higgins, 2018). These studies provided a phenomenological insight into the role of MTs in cardiovascular injury, but the detailed molecular perspective regarding their role has not yet been elucidated. In addition, other researchers have focused on tubulin posttranslational modifications, including detyrosination and tyrosination, which are currently emerging as key modulators of MTs. They demonstrated that tubulin detyrosination affected mechanotransduction in muscle cells and was crucial for load-bearing of buckling MTs during cardiomyocyte contraction (Kerr et al., 2015). Excess or reduced tubulin detyrosination was reported to affect the stiffness of cardiomyocytes, thereby contributing to cardiac dysfunction. Upregulation of tubulin detyrosination is also exhibited in patients with hypertrophic and dilated cardiomyopathies (Robison et al., 2016). These clinical links suggested that an imbalance in the levels of tubulin tyrosination and detyrosination may be a risk factor for heart failure, and could be linked to muscle dysfunctions in various other diseases. Thus, a better understanding of the posttranslational regulation of MTs might substantially change the way we perceive the roles of MTs in CVD (Magiera et al., 2018).

## Mitochondria in CVD

Mitochondria are double-membrane bound intracellular organelles responsible for energy production and regulation of cellular metabolism, and occupy a core role in eukaryotic cells, particularly cells of the cardiovascular system (Galluzzi et al., 2012, 2018; Lopez-Crisosto et al., 2017; Mehta et al., 2017). Mitochondrial dynamics (both fusion and fission), mitophagy, and homeostasis are crucial factors in the maintenance of mitochondrial integrity. Mitochondria are more prone to stress, including hypoxia and ischemia–reperfusion. Several studies have shown that accumulation of reactive oxygen species, inflammatory damage, molecular defects, and abnormal signal transduction pathways, including AMPK/ULK1, PI3K/Akt, and p38/MAPK/MAP4, lead to mitochondrial dysfunction and are at the root of numerous diseases. Alterations in the endogenous apoptotic pathway, acute exercise, impaired mitophagy, excessive or untimely mitochondrial fission or fusion, and aging are also known to lead to mitochondrial dysfunction, followed by abnormal metabolism. Alteration in metabolism leads to defects in mitochondrial biogenesis and energy supply, cell death, and calcium fluxes, which are contributing factors toward the pathogenesis of multiple cardiovascular disorders such as myocardial infarction, cardiomyopathies of various etiologies, hypertension, and atherosclerosis (Hu et al., 2014; Bravo-San Pedro et al., 2017; Suomalainen and Battersby, 2018; Zhou et al., 2018b; Wu et al., 2019a, b). In addition, impaired mitochondria have been demonstrated to actively promote inflammatory responses and cell death, thereby contributing to the pathogenesis of CVD. Based on these studies, attempts were made to develop mitochondria-targeting drugs for the treatment of cardiovascular disorders in the clinical setting. The main direction of these interventions involved mitochondrial metabolism, mitochondrial dynamics, mitophagy, calcium homeostasis, oxidative stress, inflammation, regulated cell death, and mitochondrial microRNAs. Despite extraordinary efforts spanning three decades, results have been rather dismal and a small number of effective targeted drugs have been approved for the treatment of patients with CVD (Bonora et al., 2019). Thus, molecules with optimized pharmacological characteristics and precise mechanistic insights into mitochondrial processes, along with a reassessment of the pathogenesis of CVD, are essential for the development of new drugs with clinical utility.

## Trends in MAP4-Mediated CVD

Accumulating evidence has suggested that MT and mitochondria are crucial subcellular components involved in the pathogenesis of CVD. Moreover, MAP4 is a key mediator in regulating MTs and mitochondria (Li et al., 2018, 2019; Zhang et al., 2019b). Fang et al. (2011) demonstrated that hypoxia-induced MAP4 phosphorylation in cardiomyocytes leads to MT disassembly, whereas MAP4 overexpression promotes MT polymerization leading to the stabilization of MT networks. Besides, they found that MAP4 overexpression increased viability and ATP content of cardiomyocytes under hypoxia. In addition, MAP4 was shown to inhibit the increased permeability of the mitochondrial membrane under hypoxia via regulating the interaction between dynein light chain tctex-type (DYNLT1) and voltage-dependent anion channel 1 (VDAC1) (Fang et al., 2011). Our previous study suggested that p38/MAPK affected MT dynamics and the viability of cardiomyocytes by regulating MAP4 phosphorylation under hypoxic conditions. Following hypoxia, p38/MAPK and MAP4 phosphorylation increased, with activation of p38/MAPK leading to MAP4 phosphorylation, which in turn contributed to MT disassembly and cell viability reduction. We also revealed that p38/MAPK interacts with MAP4, and thus administration of the p38/MAPK inhibitor could increase MT polymerization and enhance the viability of cardiomyocytes after hypoxia. In contrast, MKK6 (Glu), the upstream activator of p38/MAPK, was shown to reduce cell viability and promote MT disassembly under the same conditions (Hu et al., 2010). We further showed that in hypoxic cardiomyocytes, MAP4 phosphorylation was notably increased with MAP4-MT dissociation, and MAP4 translocated to mitochondria where it induced mitochondrial permeability transition pore opening and cytochrome c release followed by endogenous mitochondrial apoptosis in cardiomyocytes (Hu et al., 2014). In our recent study, we showed that MAP4 phosphorylation was evident not only in cardiac tissues in right ventricular hypertrophy in tetralogy of Fallot, but was also increased in the hearts of both myocardial infraction and transverse aortic constriction mouse models. Briefly, mice exhibiting MAP4 phosphorylation exerted age-dependent effects such as pathological cardiac remodeling, cardiomyocyte mitochondrial apoptosis and disruption, and systolic and diastolic dysfunction. As such, MT disassembly, translocation of phosphorylated MAP4, and cardiomyocyte apoptosis are deemed important factors in initiating cardiac remodeling, with the activation of p38/MAPK being suggested as the crucial upstream kinase involved in the MAP4 phosphorylation (Li et al., 2018) as shown in previous studies (Teiger et al., 1996; Choi et al., 2009; Dees et al., 2012). Interestingly, some other studies revealed that MAP4 (Ser472, Ser924, and Ser1056) phosphorylation increases in the model of pressure overload-induced cardiac hypertrophy. When these three sites were mutated to alanine and transfected into cardiomyocytes to obtain mutant MAP4 constructs, the dephosphorylated MAP4 (Ser924 and Ser1056) caused MT reorganization in the pressure overload model, similar to the hypertrophic cardiac MT phenotype, indicating that MAP4 (Ser924 and Ser1056) dephosphorylation might be the core process in mediating MT reorganization. Further, the underlying mechanism correlated with the activation of the serine/threonine phosphatase 1 and 2A, as well as the upstream p21 kinase (Cheng et al., 2010; Chinnakkannu et al., 2010). Takahashi et al. (2003) reported that MAP4 overexpression not only promoted tubulin expression and stability but also changed the characteristics of MT networks, which is considered a key factor in mediating the phenotype of the pressure overload-induced cardiac hypertrophy. Although there have been some inconsistencies in the proposed mechanisms of MAP4-mediated cardiac remodeling, this might be attributed to different models and phosphorylation sites used in these studies. Our focused sites were located in the proline-rich regions and were responsible for the longitudinal affinity between each tubulin dimer in a protofilament, whereas focused sites in other studies were located in the KXGS region, which is responsible for lateral protofilament–protofilament interactions (Katsuki et al., 1999). Even though both regions are located in the MT binding domain, the detailed functional mechanisms and resulting phenotypes may not be identical. Thus, further research is necessary to elucidate the mechanisms underlying these potential differences.

Another study revealed that the total expression of the cardiac MAP4 protein reached its highest level in the first week in neonatal rats, and then was shown to gradually reduce with aging. Likewise, MAP4 was also demonstrated to be involved in drug- and hypothermia-induced MT stability in cardiomyocytes of newborns, suggesting that the regulation of MAP4 plays an important role in MT functions during cardiac development (Webster and Bratcher, 2006). In addition, previous studies have demonstrated that MAP4 might be a potential regulator of vascular permeability. In microvascular endothelial cells, tumor necrosis-α (TNF-α)/lipopolysaccharides (LPS) induced MAP4 phosphorylation-dependent MT disassembly, followed by increased vascular permeability via activation of p38/MAPK (Li et al., 2015), providing a novel insight for MAP4 phosphorylation in regulating microvascular permeability. Furthermore, Zhou et al. (2015) found that LPS stimulation leads to disruption of MT dynamics through the activation of p38/MAPK in endothelial cells, which was shown to be blocked by a MT stabilizer. Moreover, p38/MAPK was observed to interact with MAP4 to form a complex, subsequently leading to MT disassembly, which could be suppressed by a p38/MAPK inhibitor (Zhou et al., 2015). These data indicate that MAP4 phosphorylation-dependent MT disassembly plays a key role in regulating the permeability of endothelial cells. Another study revealed that Down syndrome exhibits variable features, including heart defect, which is correlated with mitochondrial dysfunction. Overexpression of genes on chromosome-21 is responsible for the phenotypic features of Down syndrome either in a direct or in an indirect manner (Izzo et al., 2018). Our previous study showed that MAP4 phosphorylation is deemed a crucial factor in mediating mitochondrial apoptosis, morphological and functional disruption, and heart defects, which is partially similar to the mitochondrial phenotype of Down syndrome. However, whether MAP4 is involved in Down syndrome remains unclear and warrants further studies.

## Future Research Focuses

**FIGURE 1:**
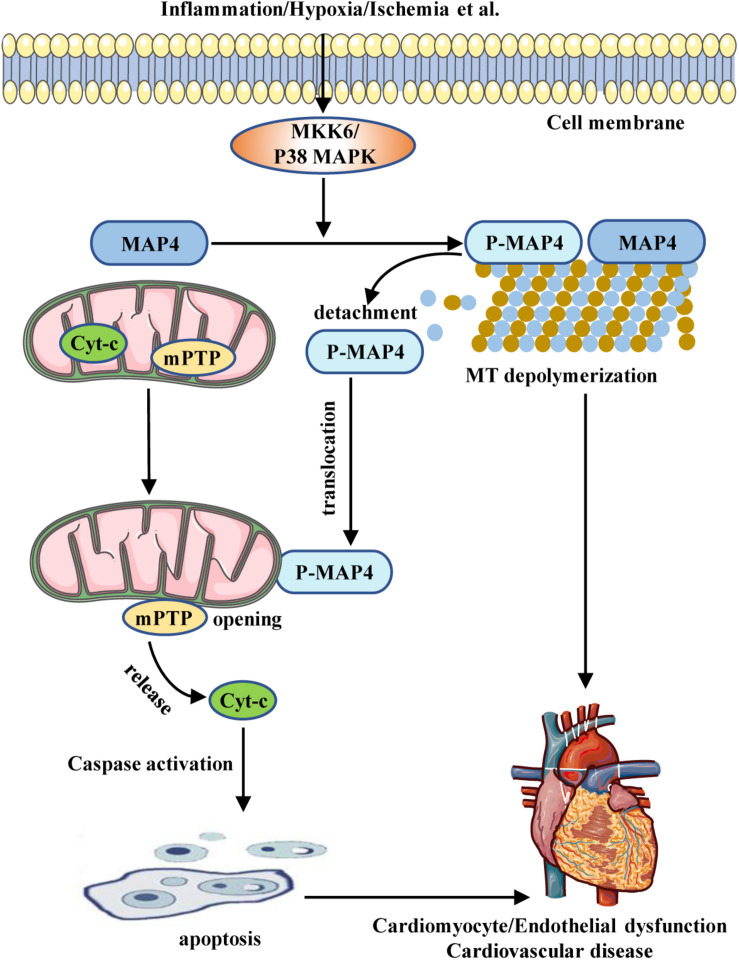
Schematic illustrating the role of MAP4 in CVD. Inflammation, hypoxia, ischemia, etc., stimulate the activation of the MKK6/p38 MAPK pathway. The activated p38 MAPK induces MAP4 phosphorylation and, sequentially, the depolymerization of MT. The phosphorylated MAP4 detaches from MT and translocates to mitochondria from cytosol, leading to mitochondrial permeability transition pore (mPTP) opening and cytochrome c (cyt-c) release, which activates caspase pathway and promotes mitochondrial apoptosis. Increased apoptosis together with MT depolymerization causes cardiomyocyte/endothelial cell dysfunction, which is involved in the pathogenesis of CVD.

Conclusively, MAP4 has been demonstrated to regulate MTs or mitochondria in cardiomyocytes and endothelial cells, both of which play a key role in the pathogenesis of CVD (Figure 1). The correlation and interactions between MAP4 and MT/mitochondria, and MT/mitochondrial-targeting drugs may provide useful tools to analyze potential MAP4-related signaling pathways or molecular interactions among p38/MAPK, septins, DYNLT1, and VDAC1. Moreover, the elucidation of their association with pathological outcomes may provide novel targets for the development of MAP4-targeting drugs. In addition, MAP4 is a unique member of MAPs, which is expressed in both vascular tissues and the heart. Recent studies have shown an important role of MAP4 in the cardiovascular system, and interventions targeting the function of MAP4 may provide protection against cardiomyocyte or endothelial cell dysfunction. For these reasons, MAP4 may serve as a potential target for pharmacological interventions in CVD.

In contrast, although there has been some progress regarding MAP4 in both basic and clinical studies, more information on MAP4, including its detailed structure, function, and regulatory mechanism, remains elusive. Thus, future studies should focus on these issues and elucidate the role of MAP4 specific to CVD both *in vivo* and *in vitro*. Meanwhile, unraveling of related pathways, molecule–molecule interactions, and the role of MAP4 in other systems may provide us with substantial evidence for the development of novel drugs targeting MAP4.

## Author Contributions

LL, QZ, XL, and YH participated in research design. JH supervised in research design. LL, QZ, XL, YH, and JH wrote or contributed to the writing of the manuscript. All authors contributed to the article and approved the submitted version.

## Conflict of Interest

The authors declare that the research was conducted in the absence of any commercial or financial relationships that could be construed as a potential conflict of interest.
